# Calculation of effective pump dose in X-ray-pump/X-ray-probe experiments

**DOI:** 10.1107/S1600577525006939

**Published:** 2025-08-21

**Authors:** Sebastião Antunes, Michal Stransky, Victor Tkachenko, Ichiro Inoue, Philip Heimann, Konrad J. Kapcia, Beata Ziaja

**Affiliations:** a Center for Free-Electron Laser Science CFEL Deutsches Elektronen-Synchrotron DESY Notkestr. 85 22607Hamburg Germany; b Institute of Nuclear Physics, Polish Academy of Sciences Radzikowskiego 152 31-342Kraków Poland; c European XFEL Holzkoppel 4 22869Schenefeld Germany; d Institute of Physics, Czech Academy of Sciences, Na Slovance 2, 182 21Prague 8, Czech Republic; e RIKEN SPring-8 Center, 1-1-1 Kouto, Sayo, Hyogo679-5148, Japan; f University of Hamburg Institute for Experimental Physics/CFEL Luruper Chaussee 149 22761Hamburg Germany; g Linac Coherent Light Source SLAC National Accelerator Laboratory 2575 Sand Hill Road Menlo Park CA94025 USA; h Institute of Spintronics and Quantum Information, Faculty of Physics and Astronomy Adam Mickiewicz University in Poznań Uniwersytetu Poznańskiego 2 PL-61614Poznań Poland; SLAC National Accelerator Laboratory, USA

**Keywords:** femtosecond studies, free-electron laser, dynamical studies, pump–probe, XFEL

## Abstract

In pump–probe experiments on solid materials performed within ultrafast X-ray science, the energy deposited by an X-ray pump pulse in the sample has a non-uniform spatial distribution. The following X-ray probe pulse then measures a volume-integrated average of contributions from the differently irradiated regions of the sample. Here we propose a scheme to calculate an effective fluence of the pump pulse such that the observable of interest calculated with the effective fluence is very close to the volume-integrated observable. This approach simplifies computational simulations of X-ray irradiated solids, which typically use periodic boundary conditions and assume a uniformly irradiated simulation box. Obtaining a prediction on a volume-integrated observable requires a significant computational effort, as it is necessary to run multiple simulations for the different exposure conditions and then perform their volume integration. The proposed scheme reduces this effort to a single calculation with the effective fluence.

## Abstract 2

A scheme is proposed to calculate an effective fluence of a spatially non-uniform pump pulse such that the observable of interest calculated with the effective fluence is very close to the volume-integrated observable. This approach simplifies computational simulations of X-ray irradiated solids.

## 1. Introduction

X-ray-pump/X-ray-probe experiments enable time-resolved studies of electronic and structural changes in materials following X-ray irradiation (Inoue *et al.*, 2021 ▸; Inoue *et al.*, 2022 ▸; Inoue *et al.*, 2023 ▸; Opara *et al.*, 2018 ▸; Pardini *et al.*, 2014 ▸; Inoue *et al.*, 2016 ▸; Ferguson *et al.*, 2016 ▸; Nass *et al.*, 2020 ▸; Hartley *et al.*, 2021 ▸; Inoue *et al.*, 2024 ▸). One of the bottlenecks in the interpretation of such measurements is the spatial non-uniformity of the pulses, usually assumed to have a Gaussian shape. This results in different regions of the investigated material being exposed to different pulse fluences. The following X-ray probe pulse then measures a volume-integrated average of the contributions originating from the differently irradiated regions of the sample (Tkachenko *et al.*, 2021 ▸; Heimann *et al.*, 2023 ▸).

Computational simulations of the X-ray irradiated samples are frequently performed in order to interpret experimental results. Such schemes typically use periodic boundary conditions. This implies that the simulation box – representing a small fraction of the irradiated material volume – is assumed to be uniformly irradiated (Medvedev *et al.*, 2013 ▸). A correct interpretation of the measurement results would require obtaining a prediction on a volume-integrated observable. This usually entails a significant computational effort, as it is necessary to run multiple simulations for the different exposure conditions and then perform their volume integration.

Here we propose an analytical scheme to calculate an effective fluence such that the observable of interest calculated for the effective fluence is very close to the volume-integrated observable. This approach requires pre-knowledge of how the observable in question depends on X-ray fluence. We demonstrate the effectiveness of the scheme on a study case: simulations performed with the code *XTANT* (Medvedev *et al.*, 2013 ▸) for a silicon face-centred-cubic crystal pumped with 50 eV photons. We obtain volume-integrated predictions for average atomic displacement and intensities of various Bragg diffraction reflections at different times. After applying a dedicated analytical scheme, a comparison of volume-integrated results with the effective fluence results is made, with very satisfactory results.

Application prospects and limitations of the scheme are discussed in the last section. The latter are illustrated with an example of a concrete experimental case (diamond pumped and probed with hard X-rays) reported by Heimann *et al.* (2023 ▸).

## 2. Definition and calculation of effective fluence

In a pump–probe experiment, a sample is irradiated first by a pump pulse, which initiates the desired electronic or structural transition in the material. The probe pulse arrives at different time instants, testing the actual state of the pump-irradiated sample. We will assume henceforth that the probing is non-invasive for the irradiated crystal (Chapman *et al.*, 2014 ▸; Yoon *et al.*, 2014 ▸; Inoue *et al.*, 2022 ▸). For simplicity, we assume that the pump and probe pulses have a similar Gaussian profile, with the same spatial spread but different peak energies. The respective fluences can be written as follows,
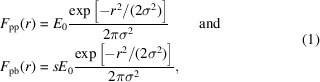
where *E*_0_ is the pump pulse energy energy and *sE*_0_ is the probe pulse energy, expressed as a fraction of the pump energy. The spatial spread is defined by σ, for both the pump and the probe pulses.

Let us now consider the observable of interest, *O*, at a given time, *t*. We assume that it depends on the pump fluence *F*_pp_ as
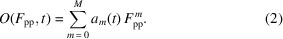
This way, we performed a standard polynomial fit to estimate the observable dependence on *F*_pp_. The polynomial order *M*, at which the series is cut, is determined by the fitting procedure described in the *Methods*[Sec sec5] section.

For argument’s sake, we will now fix the time to be the final time recorded in the experiment, *t*_final_, *i.e.O*(*F*_pp_) ≡ *O*(*F*_pp_, *t*_final_) and *a*_*m*_ ≡ *a*_*m*_(*t*_final_). The observable is probed by a radially symmetric Gaussian beam *F*_pb_(*r*). The resulting volume-integrated observable 〈*O*〉_vol_ then reads 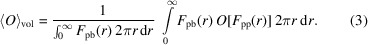
In our analysis, we assumed a uniform in-depth distribution of the absorbed beam energy in the material which, in fact, reduces the volume integration to the area integration. This uniformity assumption is only correct if the irradiated sample is thin or its thickness is comparable with the X-ray photon attenuation length (or penetration depth). The extension of our formalism, taking into account the in-depth absorption of energy (according to the Beer–Lambert law) is possible; however, it is beyond the scope of this introductory work. Performing the integration yields
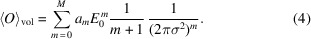
In order to determine an effective fluence value, *F*_eff_, we then equate 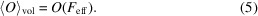
Inserting equation (4) into equation (5) yields
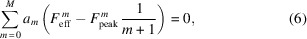
where *F*_peak_ = *E*_0_/(2πσ^2^). This equation can be solved analytically or numerically depending on the specific fluence dependence. From the multiple roots of the equation we will choose the one that is real and lies within the dose interval, [0, *F*_peak_]. For the observables we studied here, this criterion proved sufficient to identify a unique value of *F*_eff_.

We will now present how the scheme works by performing dedicated simulations of soft X-ray irradiated crystals with the *XTANT* code (Medvedev *et al.*, 2018 ▸). Beforehand, we will make a useful conversion from fluence to absorbed dose. While in experiments fluence is a natural parameter describing the integrated strength of the irradiation, theoretical simulations in general, and *XTANT* specifically, use an average absorbed dose per atom, *D*, expressed in eV atom^−1^. As discussed previously (Medvedev *et al.*, 2013 ▸; Medvedev *et al.*, 2018 ▸; Tkachenko *et al.*, 2016*a* ▸), the absorbed dose is a more universal parameter in ultrafast X-ray science, as X-ray induced transitions directly depend on the absorbed dose per atom. If the sample thickness is comparable with the attenuation length of the X-ray radiation (what we for argument’s sake assume here), the angle of X-ray incidence is normal, electron escape and surface reflectivity are negligible (*cf*. Follath *et al.*, 2019 ▸), the conversion can be readily made as 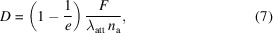
where *e* is Euler’s number, *F* is the fluence in eV cm^−2^, *n*_a_ is the number density of the irradiated sample in atoms cm^−3^ and λ_att_ (in cm) is the photon attenuation length estimated for the photon energy of the pump. We can now convert fluence to dose in equation (6),
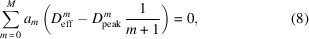
where *D*_eff_ refers to the effective dose, and *D*_peak_ refers to the peak dose, converted from *F*_peak_, using equation (7).

## 3. Calculation of effective fluence for X-ray irradiated silicon crystal

**Figure 1:**
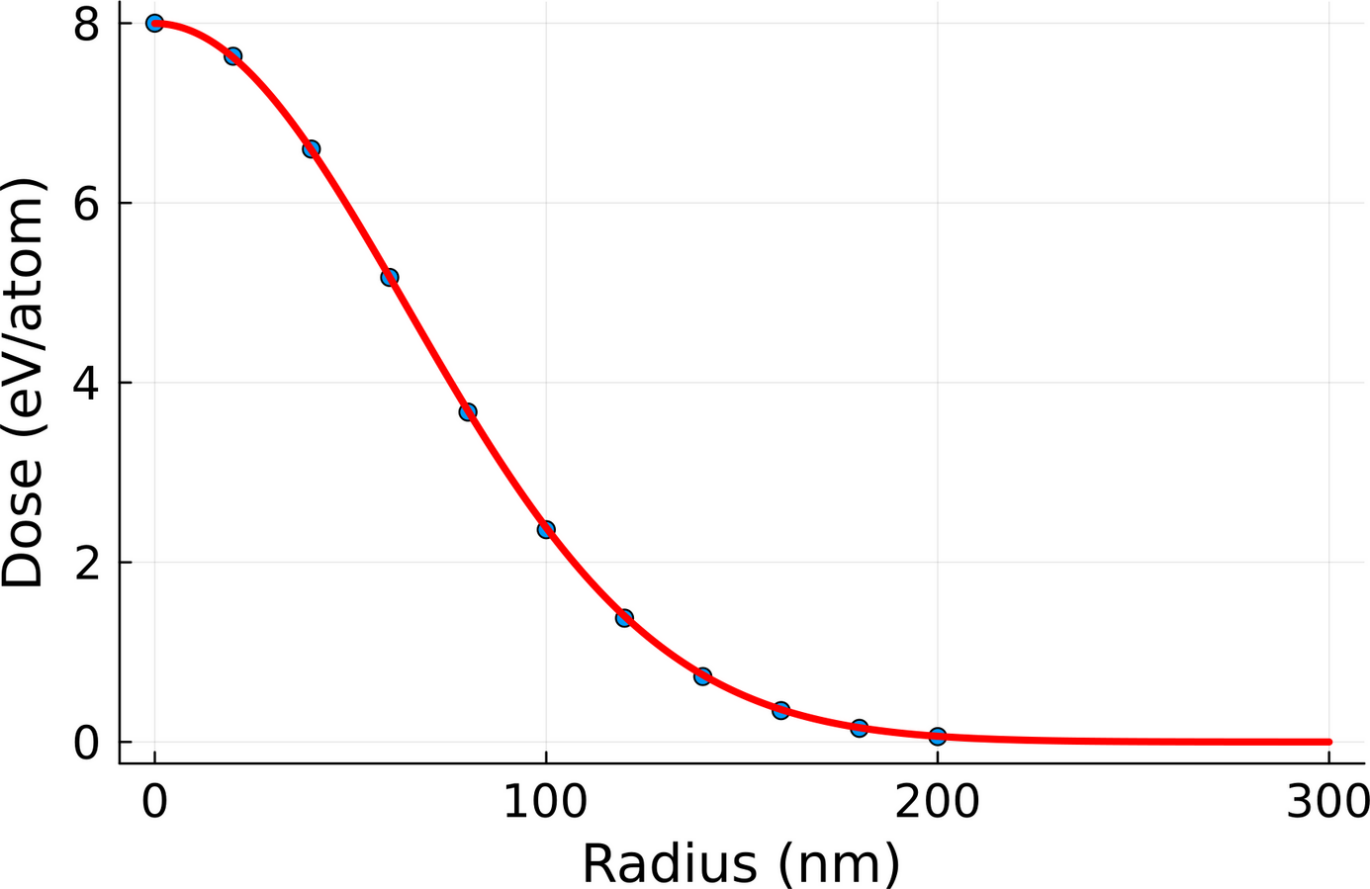
Radial distribution of the dose deposited by pump pulse (red line). Points used to run the *XTANT* simulation are marked in blue.

Below we will present the study case for the effective fluence method. Namely, we will simulate irradiation of a silicon crystal with a pump pulse of 6 fs FWHM duration and 50 eV photon energy with our code *XTANT* (Medvedev *et al.*, 2013 ▸; Medvedev *et al.*, 2015 ▸; Medvedev *et al.*, 2017 ▸; Medvedev *et al.*, 2018 ▸). Similar simulations were performed to describe experimental data by Tkachenko *et al.* (2016*b* ▸) and Medvedev *et al.* (2018 ▸), therefore, we can safely assume that the predictions are realistic enough. We will analyze structural transition in silicon initiated by a pulse of Gaussian spatial profile with σ = 63.7 nm. The peak dose deposited by the pulse is 8 eV atom^−1^ (see Fig. 1 ▸). The number of atoms in the simulation box (with periodic boundary conditions assumed) is 64. This number of atoms was chosen for expediency of the numerical simulations and should not affect conclusions regarding the correctness of the method in this purely numerical study. Since both the volume-integration and the effective-dose simulations are run for the same box size, the eventual finite size effects would act as a systematic error, affecting both simulations equally. However, if the method should be applied to describe realistic phase transitions, a convergence study with respect to the size of the simulation box should be performed. The simulation was run at times ranging from −200 fs to 100 fs, where time zero corresponds to the temporal maximum of the X-ray pump pulse.

The choice of this timescale was arbitrary as the goal of this study was to demonstrate that the effective-fluence method works, and not to study the details of phase transition in Si. The timescale chosen does not impact the conclusion that the method is sound, since both the effective-dose and volume-integrated simulations were performed for the same run time. Furthermore, as an intense pump pulse was used, 100 fs is sufficient to observe dislocations of the atoms from their equilibrium positions on the order of 10% of the interatomic distance in silicon. Concerning the divergence between the effective-dose and volume-integrated calculations at later times (close to 100 fs), the reason for it is the decreasing quality of the polynomial fit in the regime of quickly changing observables. This could be improved by extending the simulation time and fitting the coefficients once at the new final time point of the simulation (not considered here).

### 3.1. Analysis of atomic displacements

**Figure 2:**
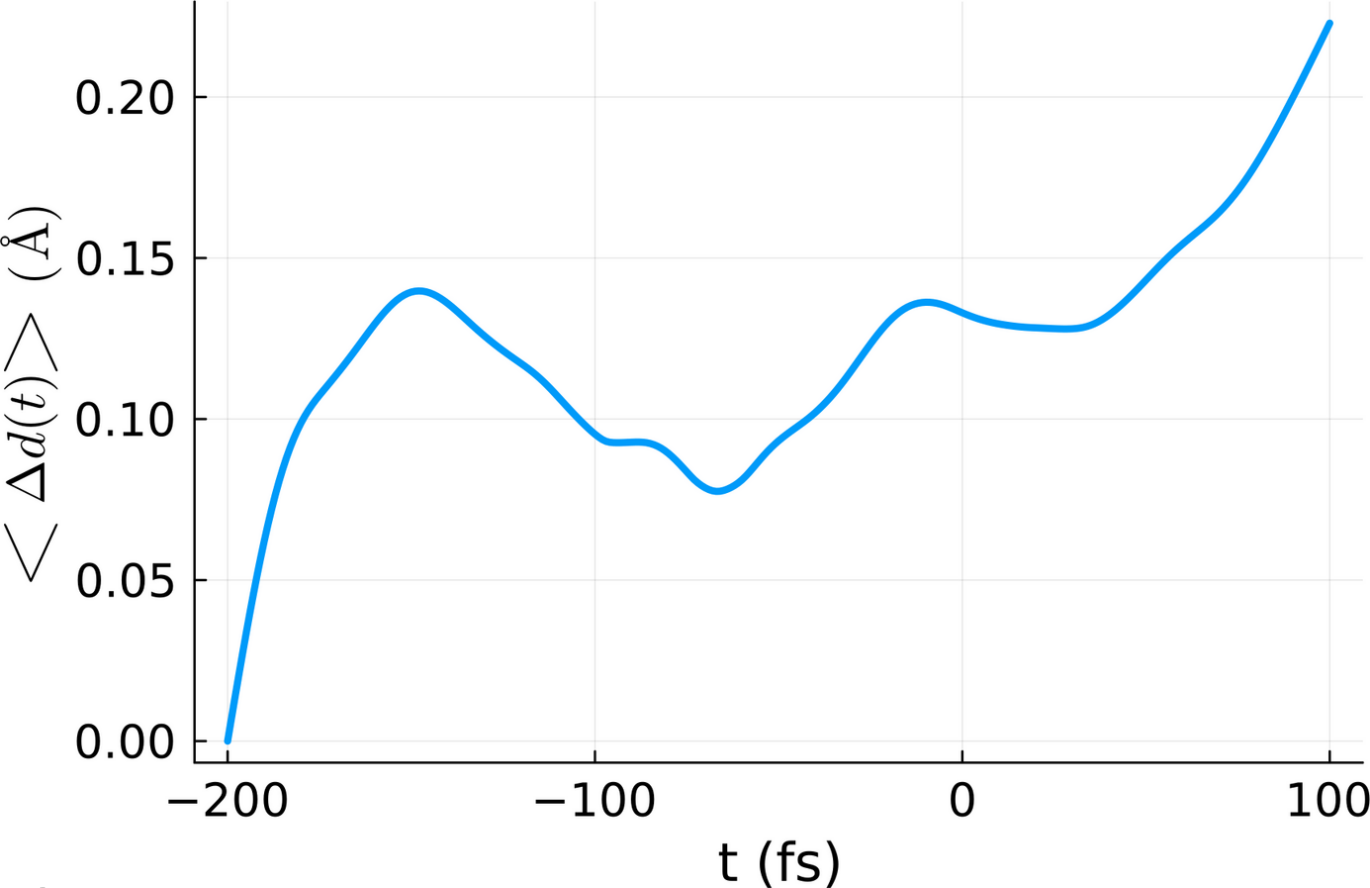
Example of average atomic displacement 〈Δ*d*(*t*)〉 for an *XTANT* simulation performed for silicon with 64 atoms in the simulation box. The other parameters were: *D* = 3.7 eV atom^−1^, pulse duration of 6 fs FWHM, and photon energy of 50 eV. The atomic displacement shows thermal oscillation before irradiation. After irradiation it starts to increase quickly.

At first, we have analyzed atomic displacements. In the periodic boundary condition framework applied here, the displacement of a single atom in a simulation box at time *t* can be defined as
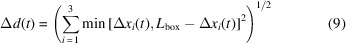

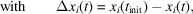
with *x*_1_, *x*_2_, *x*_3_ being the three Cartesian coordinates of an atom in the simulation box at time *t* and *L*_box_ being the cubic box size. The time instant *t*_init_ corresponds to the initial time at which the simulation was started, *i.e.* when silicon was in the ambient state (*T* = 300 K) with all of its atoms in their crystal lattice points. From the output of the *XTANT* code (for a fixed dose), we obtain the displacement as a function of time for each atom in the simulation box. Summing up the displacements of all atoms in the simulation box and dividing the sum by the number of atoms, we obtain the average atomic displacement as a function of time. Fig. 2 ▸ shows an example of average displacement obtained for a fixed dose of 3.7 eV atom^−1^.

Our in-house code *VOLINT* [similar to *XSINC* (Abdullah *et al.*, 2016 ▸; Abdullah *et al.*, 2018 ▸)] was used to predict the volume-integrated displacement, calculated for the simulation results obtained with the code *XTANT*. The simulations were performed for different dose values spanning between 0 and the peak dose of 8 eV atom^−1^. In the present case (σ = 63.7 nm), we resolved the observable spatially up to a cut-off value of 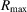 = 200 nm. We divided the range [0, 200] nm into 11 rings and considered the central dose in each ring as the input for the *XTANT* run. This is illustrated in Fig. 1 ▸. *VOLINT* then integrates the different spatial zone results for each time step and obtains 〈Δ*d*(*t*)〉_vol_, according to equation (3).

**Figure 3:**
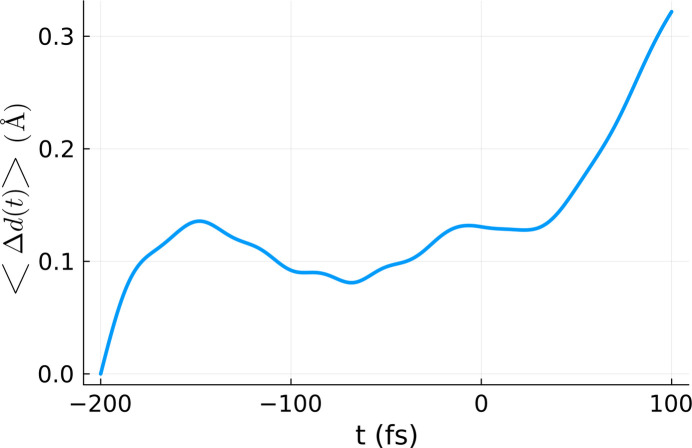
Volume-integrated and realization averaged atomic displacement 〈Δ*d*(*t*)〉_vol,real_. The integration was performed using the *XTANT* simulations performed for the doses marked in blue in Fig. 1 ▸. The calculation was performed for a silicon sample with 64 atoms in the simulation box, assuming a pulse duration of 6 fs FWHM and photon energy of 50 eV.

Since *XTANT* includes stochastic molecular dynamics simulations, it is necessary to average the simulation results over a few realizations. Previous calculations with *XTANT* (Tkachenko *et al.*, 2021 ▸; Medvedev *et al.*, 2013 ▸) showed that several realizations are enough, as averaging over electron kinetics is intrinsically performed in the code (Medvedev *et al.*, 2013 ▸; Medvedev *et al.*, 2018 ▸). Here, we performed ten realizations and averaged over them. The final output of the volume integration is presented in Fig. 3 ▸. The *XTANT* simulation starts with atoms placed in their equilibrium positions in the crystal (at zero temperature), with random velocities chosen such that the atomic temperature after thermalization should be equal to 300 K. This evolution stage can be clearly identified in Fig. 3 ▸, where, after initialization, the average atomic displacement increases up to 0.1 Å (reflecting the movement of atoms from their equilibrium positions at 0 K). Afterwards, the displacement reduces to a standard thermal atomic displacement at 300 K. After X-ray pumping, the heated crystal continuously loses its structure due to the progressing non-thermal melting process. This is reflected by an increase of atomic displacement (and the corresponding decrease of average Bragg reflection intensities).

For the calculation of the effective dose, *D*_eff_, from equation (8), it is necessary to obtain the expansion coefficients *a*_*m*_. As stated previously, we perform the estimation only for a fixed time, *t* = 100 fs, which is the final time for these simulations, in order to gain an idea about the time-dependence of the coefficients *a*_*m*_. A respective fitting procedure is required, with details provided in the *Methods* section[Sec sec5]. The optimal order for the fitting polynomial was estimated to be 5. With the polynomial coefficients calculated, we numerically solve equation (7). The solution found within our dose domain, [0, 8] eV atom^−1^, is *D*_eff_ = 4.20 eV atom^−1^.

**Figure 4:**
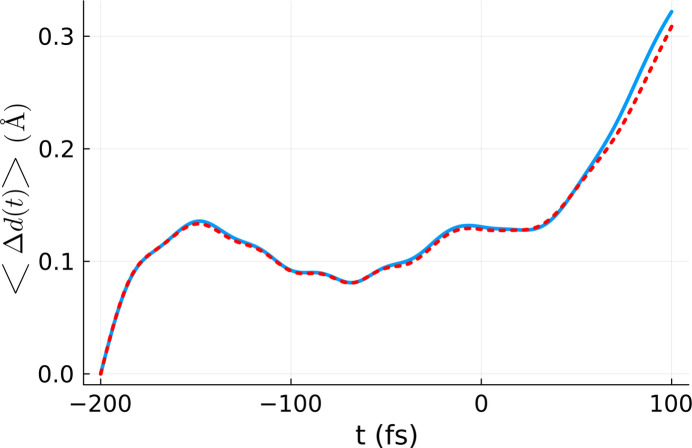
Comparison between the volume-integrated and realization averaged atomic displacement 〈Δ*d*(*t*)〉_vol,real_ (blue line) and the atomic displacement calculated for the effective dose and averaged over ten *XTANT* realizations (red line). The volume integration was performed for a silicon sample with 64 atoms in the simulation box, using the *XTANT* results for the doses marked in blue in Fig. 1 ▸ and assuming a pulse duration of 6 fs FWHM and photon energy of 50 eV. The same parameters were used for the effective dose simulation, except for the dose itself, set to *D*_eff_ = 4.20 eV atom^−1^.

In Fig. 4 ▸ we present the average atomic displacement within the simulation box, averaged over ten different *XTANT* realizations performed for the effective dose of *D*_eff_ = 4.20 V atom^−1^. It is compared with the respective volume-integrated atomic displacement from Fig. 3 ▸. The agreement between the two curves is good, which proves the correctness of the effective dose scheme. It is to be emphasized that, although the estimation of *D*_eff_ was performed at *t* = *t*_final_, there is good agreement between the average atomic displacement calculated for the effective dose and the volume-integrated one for all times. This indicates that the time-dependence of the coefficients *a*_*m*_(*t*) is practically negligible, *i.e.* the dependence of our observable on dose (fluence) is the same for all time steps.

### 3.2. Analysis of Bragg reflection intensities

We will now apply the effective dose (fluence) scheme for the intensities of various Bragg diffraction reflections. Bragg reflection intensities are directly observed in experiments and can be used, in particular, to estimate atomic displacements. The Bragg diffraction condition is: Δ**k** = **q**, where Δ**k** is the difference between the incoming and outgoing momentum and **q** is a reciprocal lattice vector described by Miller indices (Kittel, 2004 ▸). When this condition is fulfilled, the resulting structure factor associated with a given reciprocal lattice vector can be expressed as (Kittel, 2004 ▸)
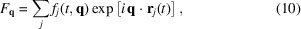
where *f*_*j*_ is the so-called atomic form factor, and 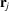 is the position of the *j*th atom in the unit cell of the crystal. The scattered (Bragg) intensity (*I*_**q**_) is then proportional to |*F*_**q**_|^2^.

**Figure 5:**
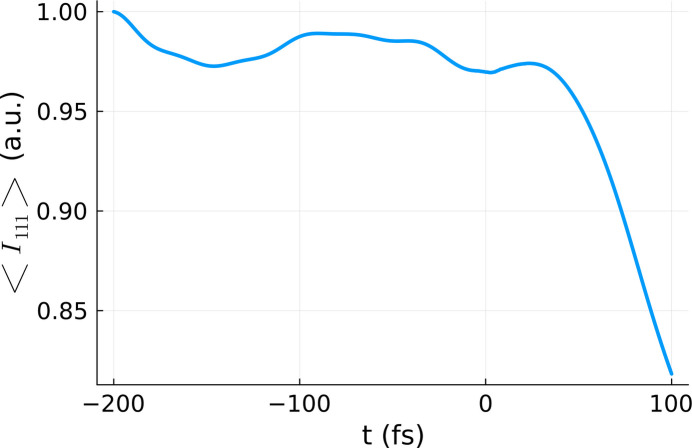
Volume-integrated 111 Bragg reflection intensity 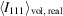. The integration was performed using the *XTANT* results for the doses marked in blue in Fig. 1 ▸. The calculation was performed for a silicon sample with 64 atoms in the simulation box, assuming a pulse duration of 6 fs FWHM and photon energy of 50 eV. The decay in the intensity observed after time zero reflects the progressing displacement of atoms.

*VOLINT* was used to predict the scattering intensity for a given Bragg reflection. *VOLINT* proceeds in the following steps: (1) performs several *XTANT* simulations; (2) from each *XTANT* simulation it obtains the actual position of each atom in the simulation box at each time step as well as the average electronic population for each atomic subshell; (3) it then distributes the number of holes randomly over the atoms in the box to obtain the average electronic population in each subshell as determined by *XTANT*; (4) afterwards, it uses the *XATOM* code (Inhester *et al.*, 2023 ▸; Jurek *et al.*, 2016 ▸; Son *et al.*, 2011 ▸) to calculate atomic form factors for all atomic species and electronic configurations; (5) it calculates *F*_q_(*t*) with equation (10); (6) finally, it performs realization averaging and then volume integration of *I*_q_(*t*) to obtain the respective (relative) Bragg signal, according to equation (3). An example of volume-integrated Bragg reflection intensity obtained with *VOLINT* is presented in Fig. 5 ▸.

**Figure 6:**
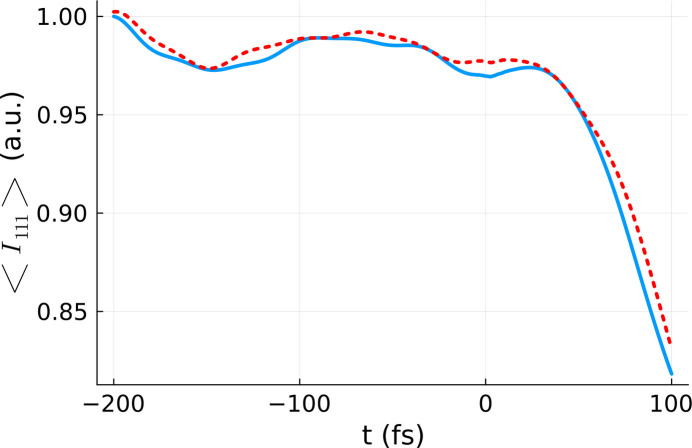
Comparison between the volume-integrated Bragg reflection intensity 〈*I*_111_(*t*)〉_vol,real_ (blue line), and the Bragg reflection intensity obtained for the effective dose 〈*I*_111_(*t*)〉_rel_ averaged over 50 *XTANT* realizations (red line). The volume integration was performed for a silicon sample with 64 atoms in the simulation box, using the *XTANT* results for the doses marked in blue in Fig. 1 ▸, and assuming a pulse duration of 6 fs FWHM and photon energy of 50 eV. The same parameters were used for the effective dose simulation, except for the dose itself, set to *D*_eff_ = 4.51 eV atom^−1^.

The same procedure as above was applied to calculate the respective *D*_eff_ for the intensity of the 111 reflection (for details see the *Methods* section[Sec sec5]). A comparison between the 111 reflection obtained for the effective dose and the volume-integrated 111 reflection is presented in Fig. 6 ▸. Again, we can see that the two curves resemble one another closely, validating our approach. An increase of the realization number to 50 realizations was carried out in order to improve the statistics. Both curves reflect thermal oscillations within silicon crystal before time zero and rapid atomic dislocation after time zero. The latter results in the Bragg intensity dropping.

Again, we note that, although the fitting of *D*_eff_ was performed at *t* = *t*_final_, there is good agreement between the effective dose and the volume-integrated curves at all times. This indicates that the time-dependence of the polynomial coefficients is practically negligible, *i.e.* the dependence of our observable on dose (fluence) is the same for all time steps.

**Figure 7:**
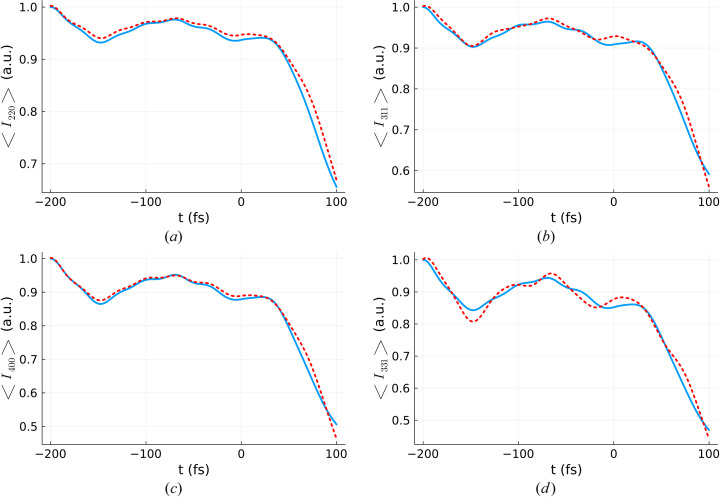
Comparison between volume-integrated Bragg reflection intensities (blue) and the Bragg reflection intensity for the appropriate effective dose, averaged over ten *XTANT* realizations (red line) for (*a*) 〈*I*_220_〉, (*b*) 〈*I*_311_〉, (*c*) 〈*I*_400_〉 and (*d*) 〈*I*_331_〉. Volume integration was performed for a silicon sample with 64 atoms in the simulation box, using the *XTANT* results for the doses marked in blue in Fig. 1 ▸, and assuming a pulse duration of 6 fs FWHM and photon energy of 50 eV. The same parameters were used for the effective dose simulation, except for the dose itself, that can be found in Table 1 ▸.

The same search procedure for an effective dose was repeated for other diffraction reflections, namely for the 311, 220, 400 and 331 reflections (see Fig. 7 ▸). The procedure needs to be repeated since different Bragg reflections have different sensitivity to atomic displacements. They will all decay as the atoms move away from their equilibrium positions, however, not in the same way, because they probe different directions of local order. As the crystal melts, it is expected that some bonds will break faster than others, creating anisotropies which make different Bragg reflections respond differently. Table 1 ▸ summarizes the predicted effective doses, both for all the Bragg reflections analyzed as well as for the average atomic displacement.

All effective doses are found within a narrow range of 4 to 4.5 eV atom^−1^, with a maximum variation of approximately 12%. This consistency indicates that the range of effective doses accurately captures the dynamics occurring in the sample during the structural transition. Conversely, if we were to calculate the dose from the so called ‘average’ fluence obtained by dividing the total beam energy over the focal area (up to the FWHM), we would obtain a value of 11.5 eV atom^−1^ for the same pump profile (see Fig. 1 ▸). Following the above-described procedure, since the full beam energy is assumed to be deposited in the FWHM focus, the relation 〈*F*〉 = 1.442*F*_peak_ will hold for any Gaussian pulse. If this average value were used in constant dose simulations, the results would differ significantly from the experimental observations.

## 4. Discussion

### 4.1. Non-Gaussian beams

The current analysis does not explore a general case of any (non-Gaussian) beam profile, as the scope of the paper is to introduce the idea of an effective fluence and to illustrate it on a typical example of a beam profile. The assumption that an X-ray beam has a spatial Gaussian profile is a frequent approximation applied during the analysis of various X-ray free-electron laser pump–probe experiments (*e.g.* Abdullah *et al.*, 2016 ▸). One-dimensional analysis can then be sufficient to obtain effective fluence. However, the effective-fluence methodology itself is not limited to the Gaussian case. If we can perform the fluence scan as proposed by, for example, Chalupský *et al.* (2013 ▸), the effective fluence can still be evaluated numerically by solving equation (5). This analysis will then be much more complex (multi-dimensional). However, strong shot-to-shot variations of the beam profile cannot be treated accurately. Predictions for the shot-averaged (Gaussian) pulse profile can then only be obtained.

### 4.2. Scaling properties

An interesting property of equation (8) is that it only depends on the peak dose, *F*_peak_, and the fitting coefficients, *a*_*m*_. If both *E*_0_ and σ would be rescaled to keep *F*_peak_ unchanged (and, as a consequence, *D*_peak_ as well), the effective dose value *D*_eff_ should remain the same.

**Figure 8:**
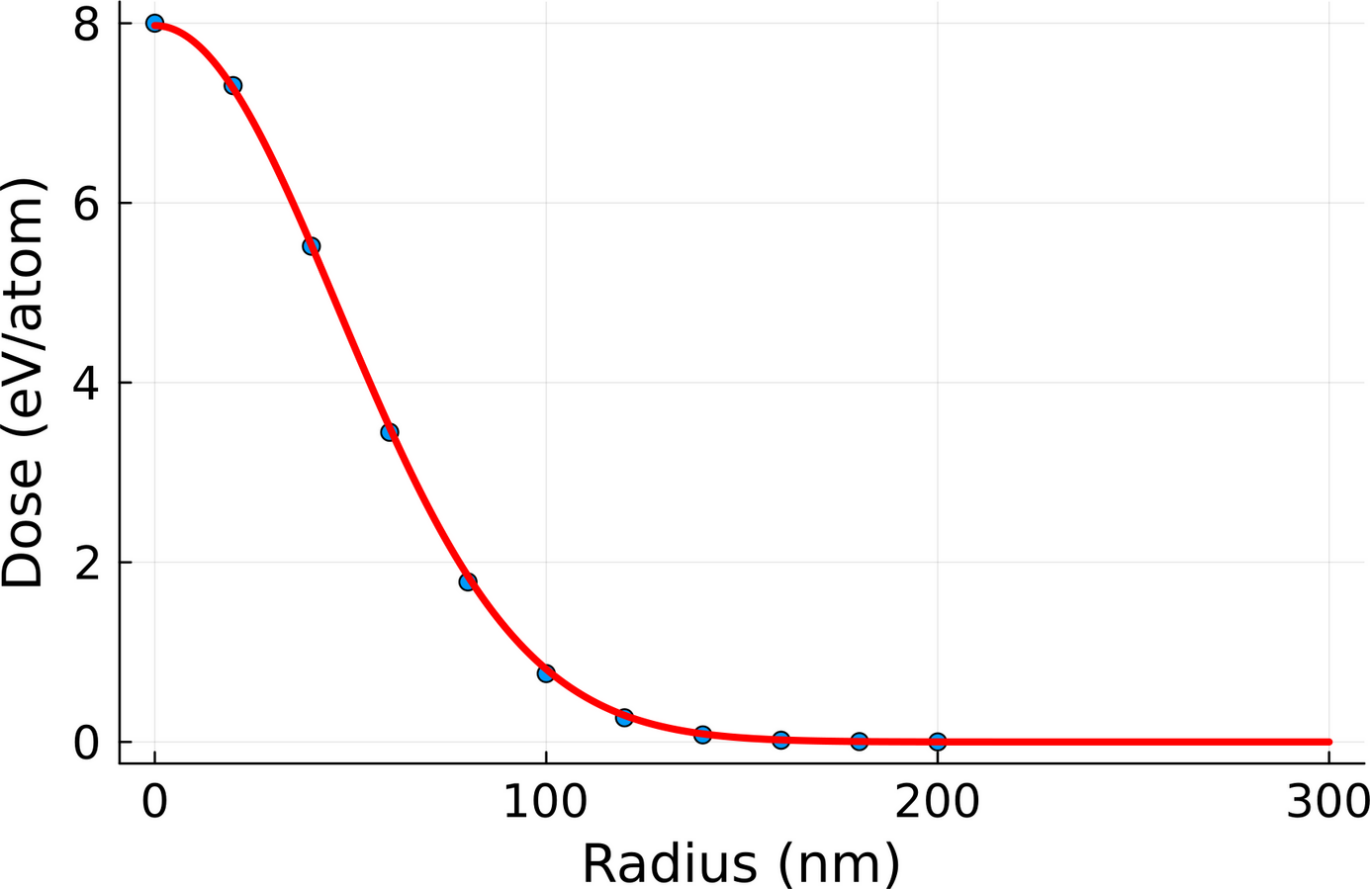
Radial distribution of the ‘rescaled’ pump pulse (red line). The points used for *XTANT* simulations are marked with blue dots.

Following this line of reasoning, we performed a simple check of the rescaling property. Namely, we reduced the total energy, *E*_0_, by half and changed σ to 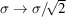. The peak fluence is then the same as before. In Fig. 8 ▸ the new radial profile of the pump pulse is shown, along with the discrete points used to perform volume integration.

**Figure 9:**
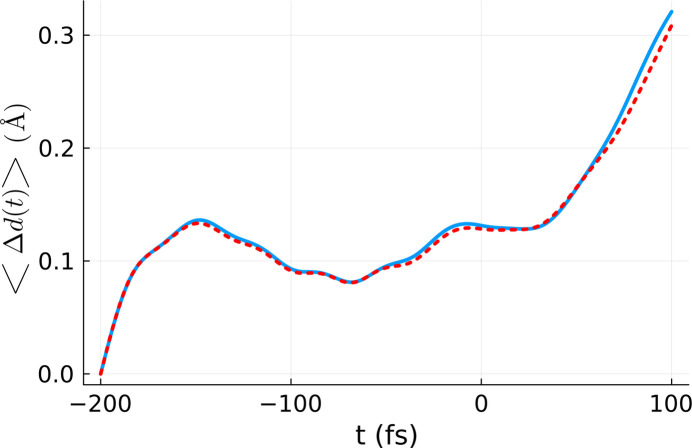
Comparison between the volume-integrated atomic displacement 〈Δ*d*(*t*)〉_vol,real_ (blue line) and the atomic displacement calculated for the effective fluence averaged over 50 *XTANT* realizations (red line). The volume integration was performed for a silicon sample with 64 atoms in the simulation box, using the *XTANT* results for the doses marked in blue in Fig. 8 ▸, and assuming a pulse duration of 6 fs FWHM and photon energy of 50 eV. The same parameters were used for the effective dose simulation, except for the dose itself, set to *D*_eff_ = 4.20 eV atom^−1^.

**Figure 10:**
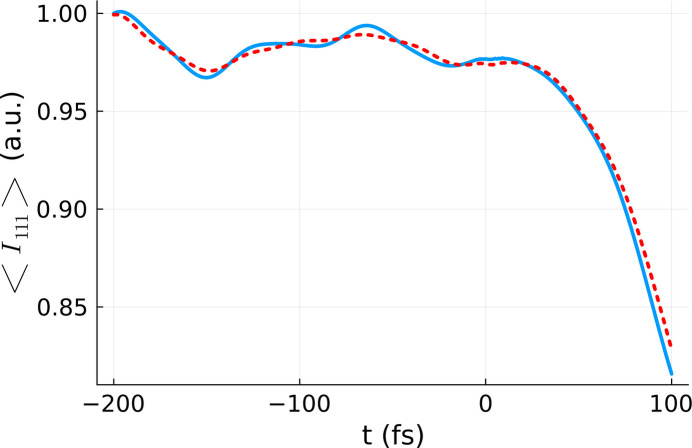
Comparison between the volume-integrated Bragg reflection intensity 〈*I*_111_(*t*)〉_vol,real_ (blue line) and the Bragg reflection intensity calculated for an effective dose averaged over 50 *XTANT* realizations (red line). The volume integration was performed for a silicon sample with 64 atoms in the simulation box, using the *XTANT* results for the doses marked in blue in Fig. 8 ▸, and assuming a pulse duration of 6 fs FWHM and photon energy of 50 eV. The same parameters were used for the effective dose simulation, except for the dose itself, set to *D*_eff_ = 4.51 eV atom^−1^.

In Fig. 9 ▸ a comparison between the volume-integrated average atomic displacement calculated at the new irradiation conditions and the results for *D*_eff_ = 4.20 eV atom^−1^ averaged over ten *XTANT* realizations are shown. The agreement between both predictions is quite good, of a quality similar to that obtained in Fig. 4 ▸, *i.e.* for the same *D*_peak_ but at different irradiation conditions. We also analyzed the time-dependent intensity of the 111 Bragg reflection presented in Fig. 10 ▸ with the same conclusion. These findings confirm the scaling feature of equation (8).

### 4.3. Effect of ballistic electrons

There are some limitations of the method; for example, it is assumed that the energy deposited by the X-ray pulse stays within the beam focus. This is true if the range of ballistic electrons released by the X-ray pump pulse is much smaller than the beam focus. Unfortunately, this is not the case when tightly focused hard X-rays are used for pumping, as the example presented below shows. Therein, experimental data are used to demonstrate the failure of the effective fluence method in this particular case. Still, a possible extension of the method by taking ballistic transport into account when estimating the effective fluence can be considered.

Heimann *et al.* (2023 ▸) reported results from a hard X-ray pump/hard X-ray probe experiment. The target was solid-density diamond that underwent partial graphitization on an ultrafast time scale. To track the transition, time-resolved intensities of several Bragg reflections were measured. The experiment was performed under different irradiation conditions. To illustrate the limitations of our method we will use one specific irradiation case for which the parameters are summarized in Table 2 ▸. Therein, *E*_0_ is the total pump pulse energy, *w*_*x*/*y*_ the 1/*e* width of the pulse, and λ_e_ the range of electrons released by 7 keV pump pulse photons. Note that in the paper by Heimann *et al.* (2023 ▸) a mistake was made and the range of electrons was quoted for an electronic cascade triggered by 8 keV and not by 7 keV photons.

To calculate the average absorbed dose, the following formula was proposed, correcting for fast electronic escape from the focal region, 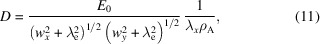
where ρ_A_ is the atomic density, and λ_*x*_ is the X-ray penetration depth for the pump’s specific photon energy. In our calculations, we assume that the pump profile is a radially symmetric Gaussian. Therefore, we apply here the approximation 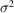

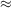

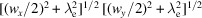
, and with it calculate *D*_peak_ ∝ *E*_0_/(2πσ^2^) for the radial Gaussian profile.

**Figure 11:**
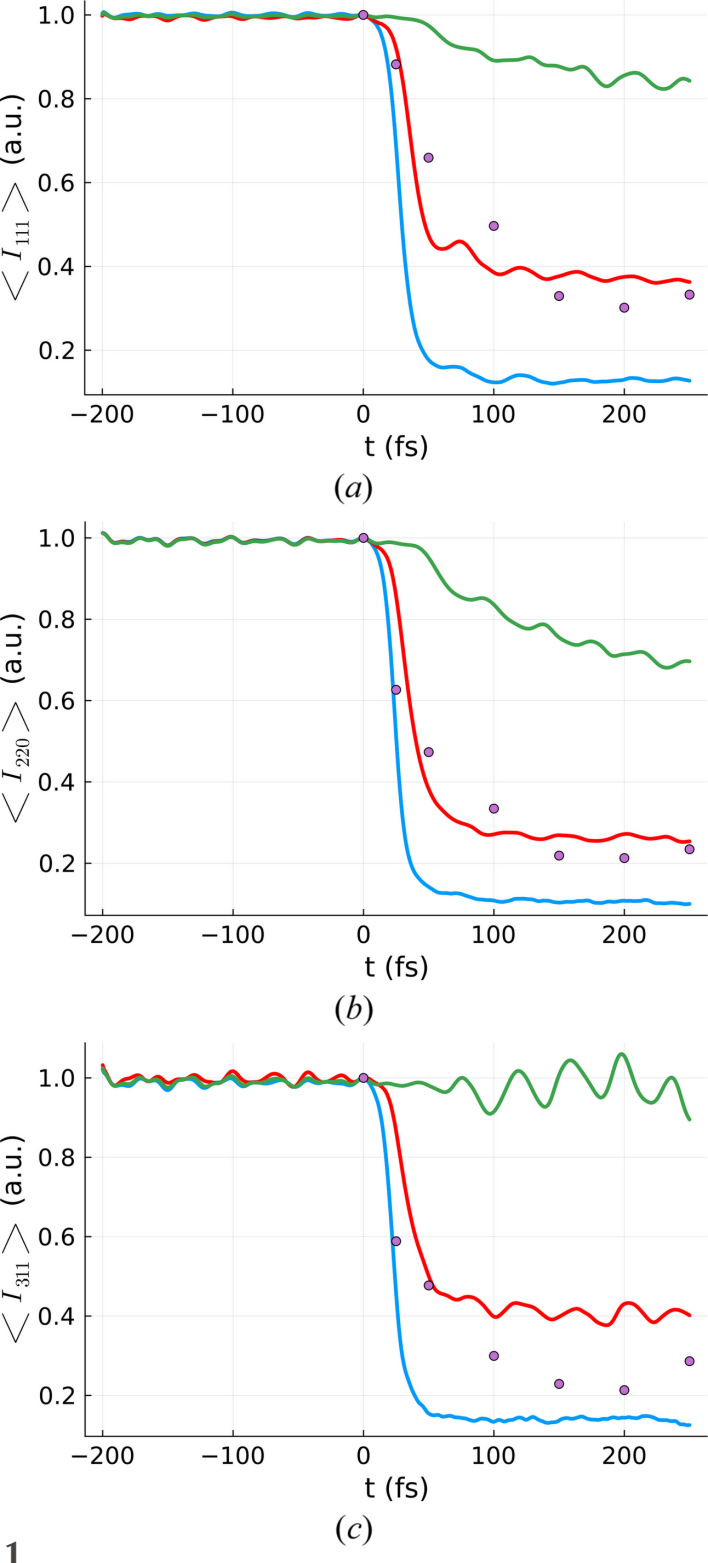
Volume-integrated Bragg reflection intensity for the irradiation conditions from Table 2 ▸ compared with the respective experimental data for (*a*) 〈*I*_111_(*t*)〉_vol,real_, (*b*) 〈*I*_220_(*t*)〉_vol,real_ and (*c*) 〈*I*_311_(*t*)〉_vol,real_. The volume integration was performed for a diamond sample, using *XTANT* code, and assuming 7 keV photons, pulse duration of 6 fs FWHM, and three different doses: 22.8 eV atom^−1^ (blue), 7.6 eV atom^−1^ (red) and 3.6 eV atom^−1^ (green). The magenta dots represent the experimental data points from Heimann *et al.* (2023 ▸).

Knowing *D*_peak_ and σ we can perform a set of simulations needed for volume integration, similarly to what we did in the previous sections. The results of these efforts are shown in Fig. 11 ▸ for the tightly focused case and the diffraction reflections 111, 220 and 311. The results with the peak dose (calculated with the method described above) are in green. As can be seen, they underestimate the Bragg reflection decay in all cases. As an improvement, the peak dose was recalculated without correcting for electronic escape (λ_e_ = 0; blue line). In this case, the Bragg reflection decay was overestimated. Finally, a peak dose was found such that the experimental and theoretical predictions would fall in the same range (orange line).

The volume integration fails to properly describe the observable and an *ad hoc* fitting procedure is required (still volume-integrated but with a peak dose not directly related to the experimental parameters). This discrepancy is probably related to the electron cascade size being comparable with the focal spot size, which also introduces a time dependence into the problem. As energy is deposited according to the pump profile, it also starts to spread out due to high-energy ‘ballistic’ electrons. Both effects happen simultaneously during the pump pulse. The electronic transport continues for a few femtoseconds afterwards.

This hypothesis is supported by the fact that the peak dose calculated with λ_e_ (representing an immediate escape of the hot electrons) gives us the upper limit for the Bragg reflection decay while the peak dose calculated without λ_e_ (the other extreme, representing the case when electrons never escape from the focus) gives us the lower limit. Since none of the limiting cases, based on actual experimental parameters, provide a good fit to the data, no effective dose calculation was attempted.

### 4.4. Summary

Our results indicate that the effective dose method can be used for simulations of pump–probe experiments, replacing the costly volume-integration scheme. Here, we tested this method on atomic displacements and Bragg reflection intensities. However, the method can also be applied to other volume-integrated observables, provided their fluence (dose) dependence can be approximated with a polynomial fit. We believe that this study offers a computationally efficient and rigorous method to estimate the effective fluence (dose) for volume-integrated experimental observables. Importantly, we find that the effective dose better represents the sample dynamics than the average dose – total beam energy divided by the focal area (up to the FWHM) – commonly used in experiments. Using the average dose in simulations can lead to significant discrepancies from experimental results, underscoring the relevance of the effective dose approach introduced here. Nonetheless, a full exploration of the prospective applications of this method goes beyond the scope of this introductory work.

## 5. Methods

### 5.1. Atomic displacement as a function of dose

As explained before, in what follows we fix time to *t*_final_ = 100 fs. The fitting procedure is applied to the pairs 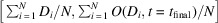, where *N* is the number of different *XTANT* realizations being averaged over (here, *N* = 10). In what follows we will study the average atomic displacement 〈Δ*d*(*t*_final_)〉_real_.

Since we do not know *a priori* the order of the polynomial dependence of 〈Δ*d*(*t*_final_)〉_real_, the fits are performed for polynomials of increasing order. To avoid overfitting, we also calculate 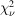, *i.e.* the reduced χ^2^, and 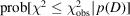, *i.e.* the probability of obtaining a χ^2^ value at least as high as the observed.

Following Taylor (1997 ▸), we define
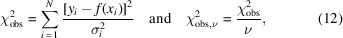
where *y*_*i*_ are data points used for the fit with the uncertainty 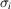 = 
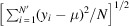
, where μ is the average data value obtained from all *XTANT* realizations for a given dose. *f*(*x*_*i*_) is the value predicted by the fit, and ν is the number of degrees of freedom. For linear models, ν = *N* − (*n* + 1), with *n* being the degree of the fitted polynomial. If our data points are independent and normally distributed then 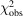 ≃ 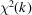. With the well known probability density function for *k* degrees of freedom, we can then write 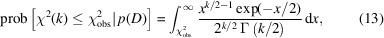
where Γ is the gamma function and 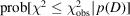, as stated above, is the probability of obtaining a test statistic at least as high as the observed one. This implies that, the higher the probability, the better the fit, and vice-versa.

**Figure 12:**
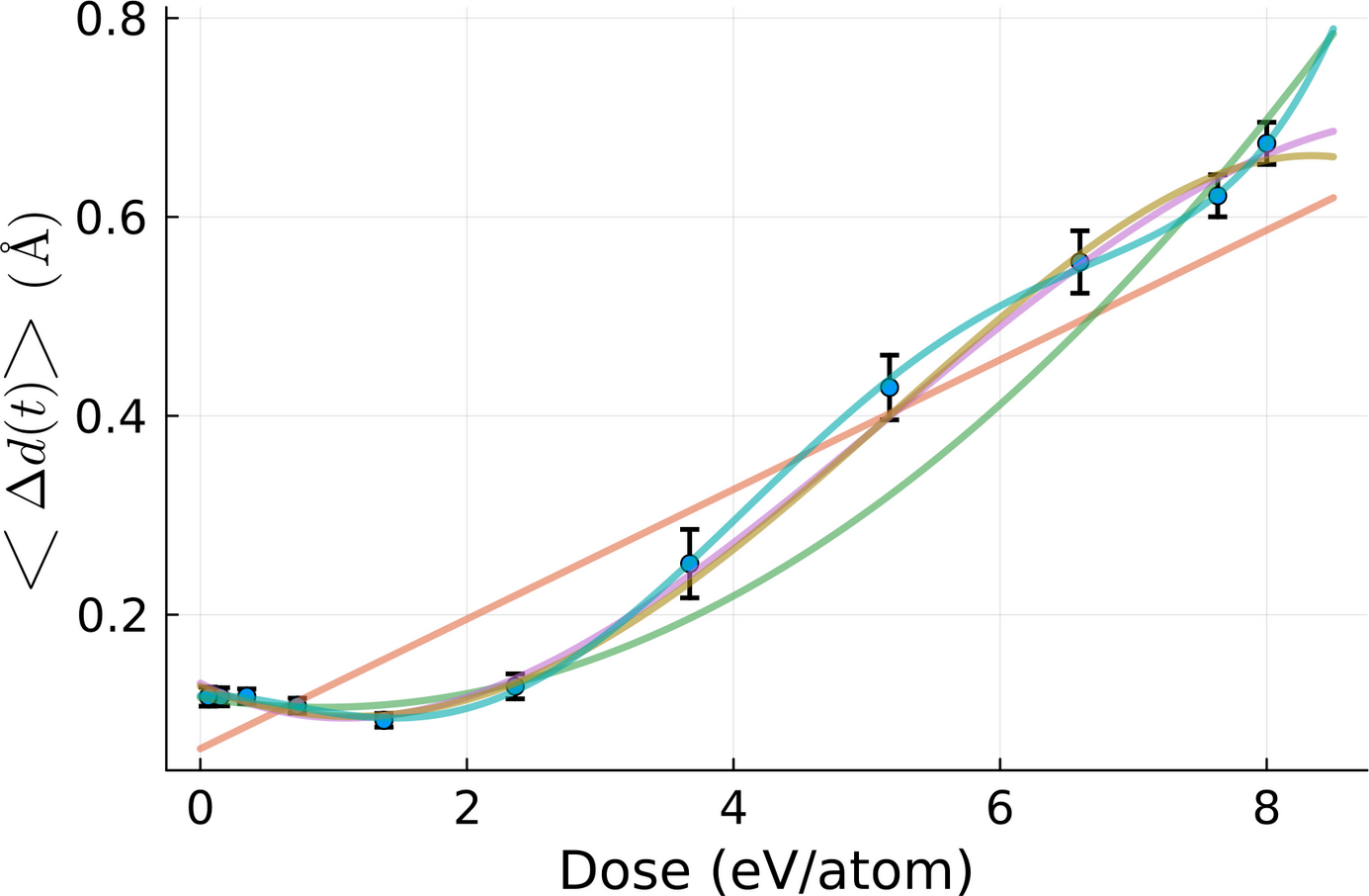
Polynomial fits of various orders applied to the data set 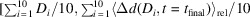], obtained from the simulations used to volume integrate the result in Fig. 3 ▸. The fits predict the dose dependence of our observable, needed for *D*_eff_ calculation.

**Figure 13:**
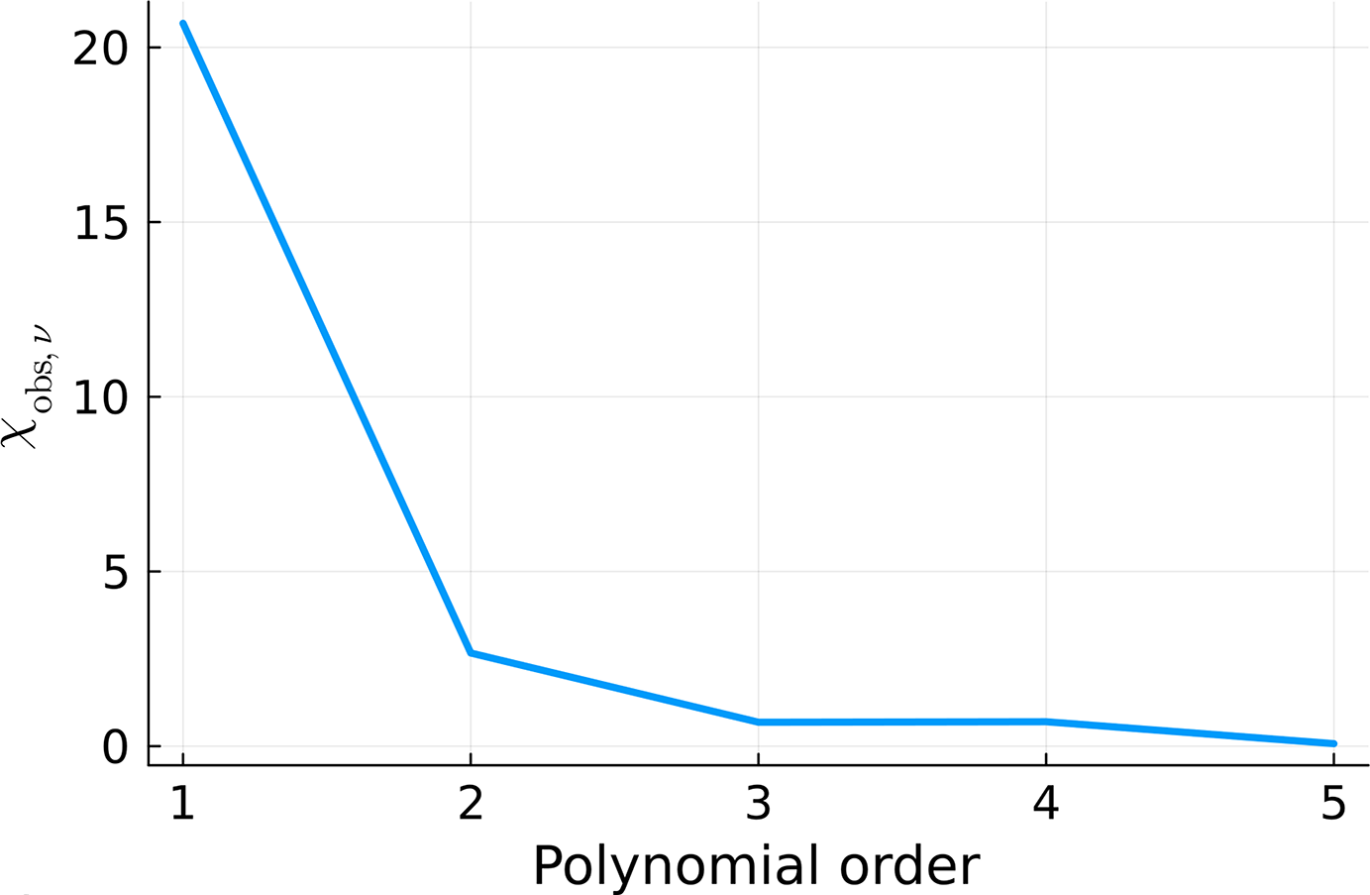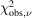 calculated for the fits displayed in Fig. 12 ▸.

We performed six fits with increasing polynomial order. They are shown in Fig. 12 ▸. We used the gradient descent method for minimizing equation (12). 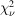 and 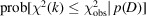 were also calculated for all of the fits, according to equations (12) and (13), respectively, and plotted in Figs. 13 ▸ and 14 ▸. They consistently indicate that the quality of the fit significantly increases with the increasing polynomial order until we reach fifth order. Afterwards, the fitting quality stabilizes. It does not change when we continue to increase the number of degrees of freedom. This is a signature of overfitting (Bishop, 2006 ▸).

Note that the fitting in Fig. 12 ▸ was performed for all fluence points, since volume integration was performed for comparison, and we had all these points available anyway. In order to test the robustness of the method in respect of the number of fluence points, we redid the fit in Fig. 12 ▸, using only six out of the 11 original fluence points, *i.e.* omitting every second point. This is the minimal number of data points needed to perform a fifth-order polynomial fit. After solving equation (8) for the new set of *a*_*m*_ coefficients, we obtained the effective dose of 4.18 eV atom^−1^ for the average atomic displacement, whereas the dose obtained from the full set of fluence points was 4.2 eV atom^−1^, *i.e.* the minimal set of six fluence points (needed to perform a fifth-order polynomial fit) was sufficient to estimate the effective dose with good accuracy. Also, in the cases discussed in this study, coefficient fitting at each time step was not necessary, as all coefficients turned out to be time-independent. However, this may not always be the case.

### 5.2. Bragg reflection intensity as a function of dose

**Figure 15:**
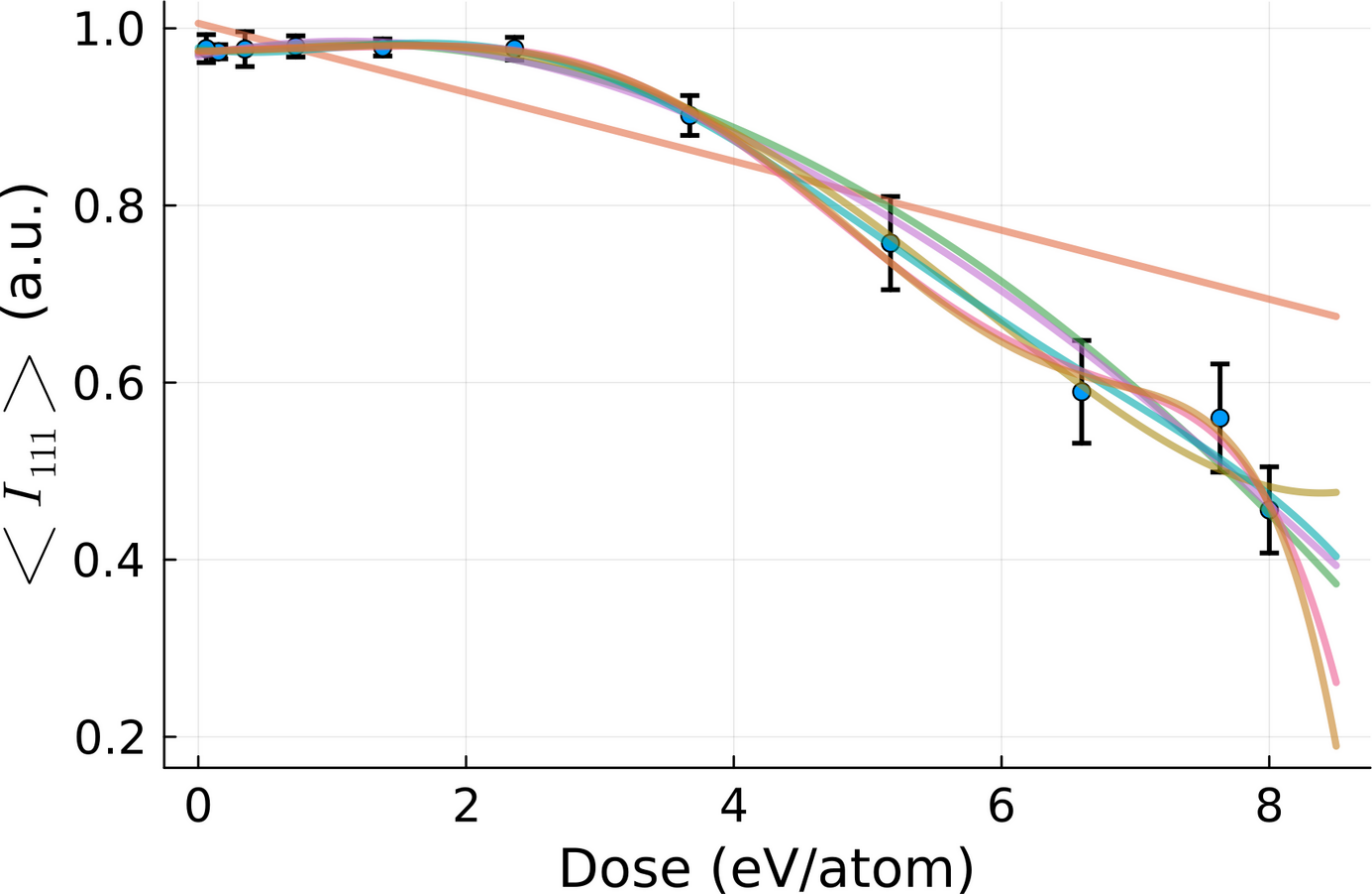
Polynomial fits of different orders performed for the data set [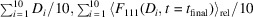], obtained from the simulations used to volume integrate the result in Fig. 5 ▸.

**Figure 16:**
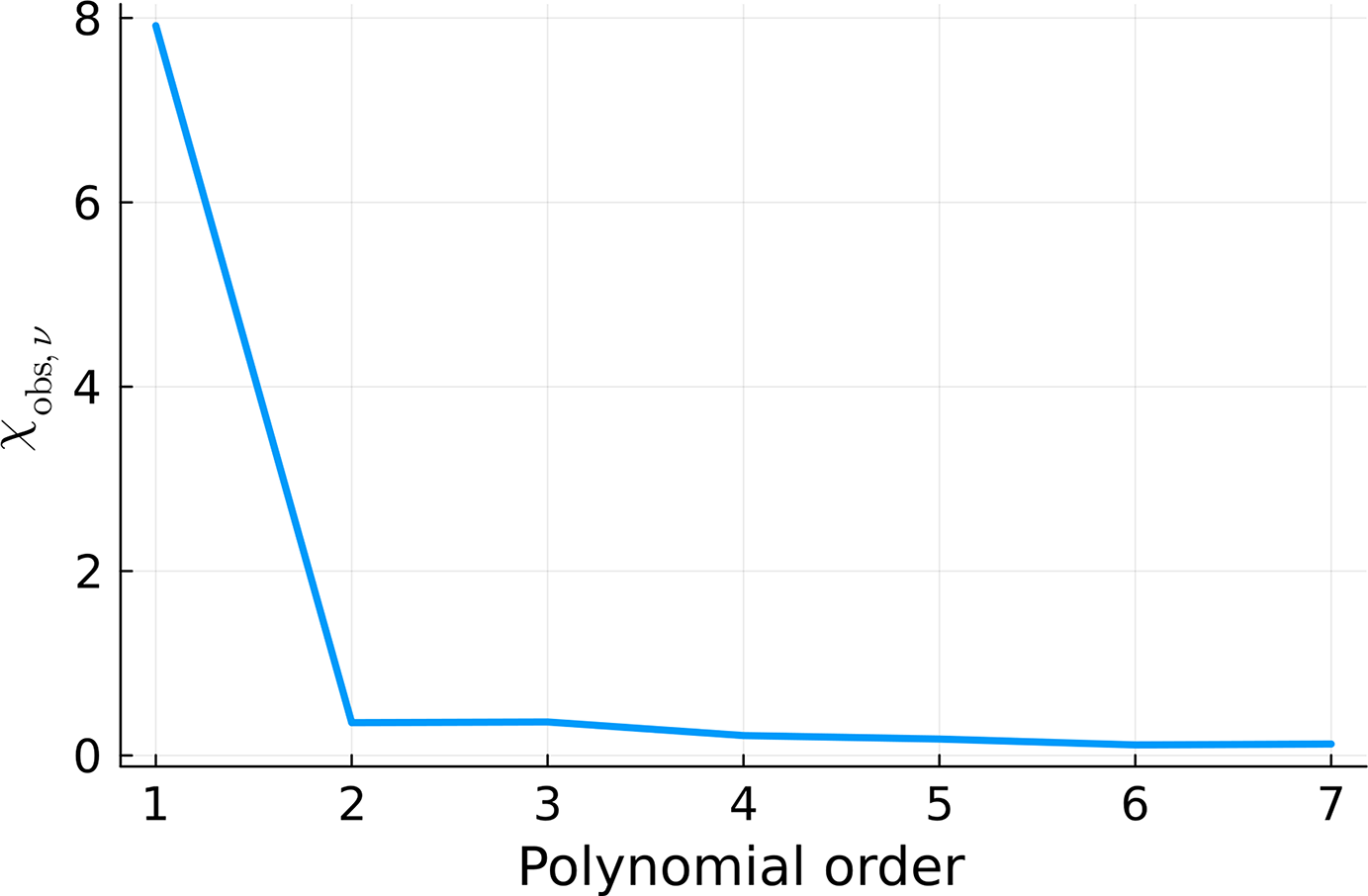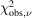 calculated for the fits displayed in Fig. 15 ▸.

The same method as above was used to study the dose dependence of the 111 Bragg reflection intensity. We performed polynomial fits of increasing order to the respective data set. In Fig. 15 ▸ the data set and their fits are shown. Note that the error bars for the Bragg signal are significantly larger than for the atomic displacement case (*cf*. Fig. 12 ▸).


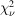
 and 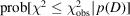 for all the fits are depicted in Figs. 16 ▸ and 17 ▸, respectively, as a function of polynomial order. They indicate that the optimal fit to represent the dose dependence of our observable is of sixth order.

As before, we use the estimated polynomial coefficients to solve equation (8). In the desired dose range of the [0, 8] eV atom^−1^ range, we obtain the solution *D*_eff_ = 4.51 eV atom^−1^. The same analysis was performed for other Bragg reflection intensities discussed in this study.

## Figures and Tables

**Figure 14:**
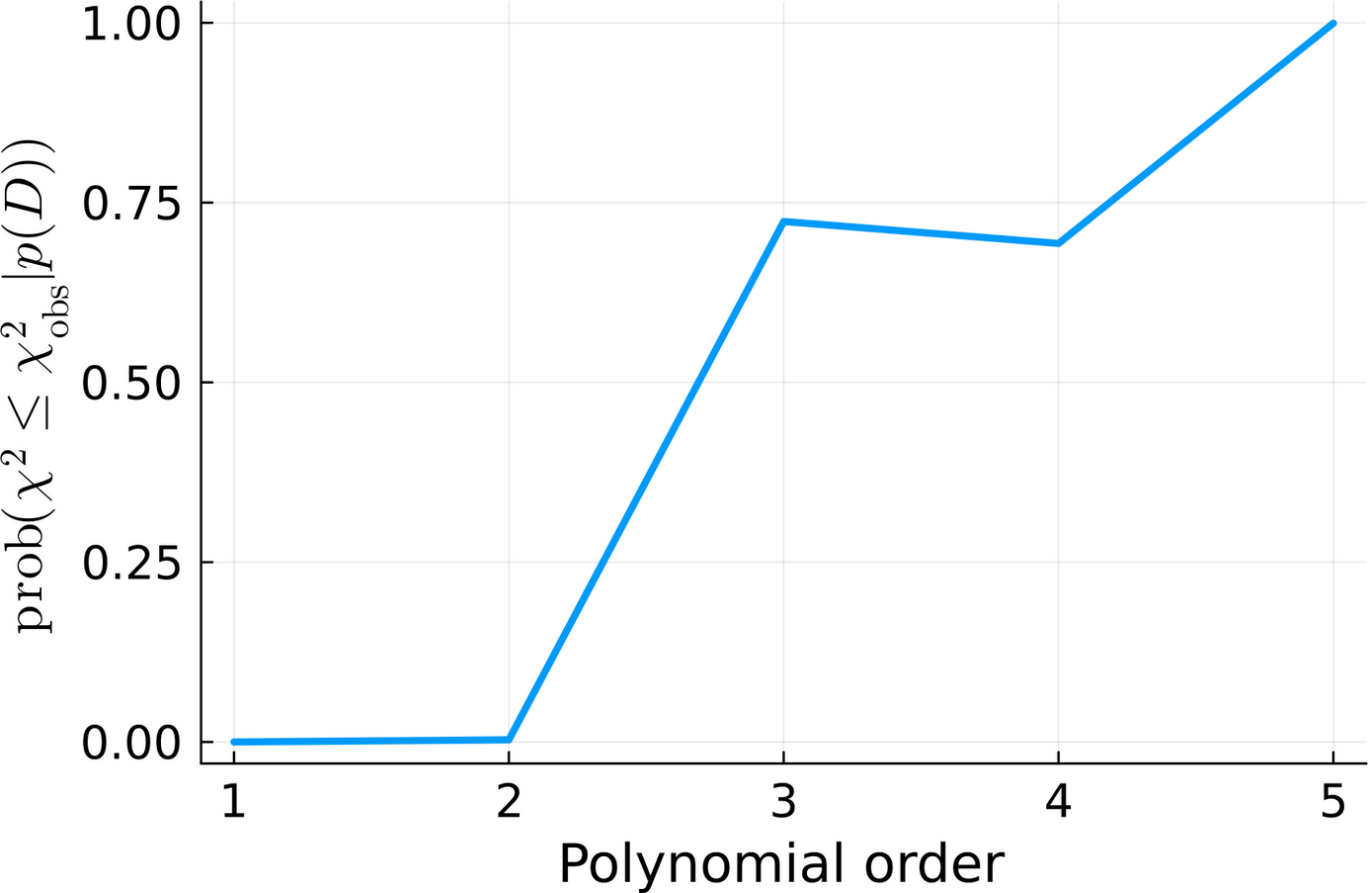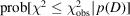 from equation (13) calculated for the 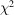 = 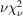 values present in Fig. 13 ▸.

**Figure 17:**
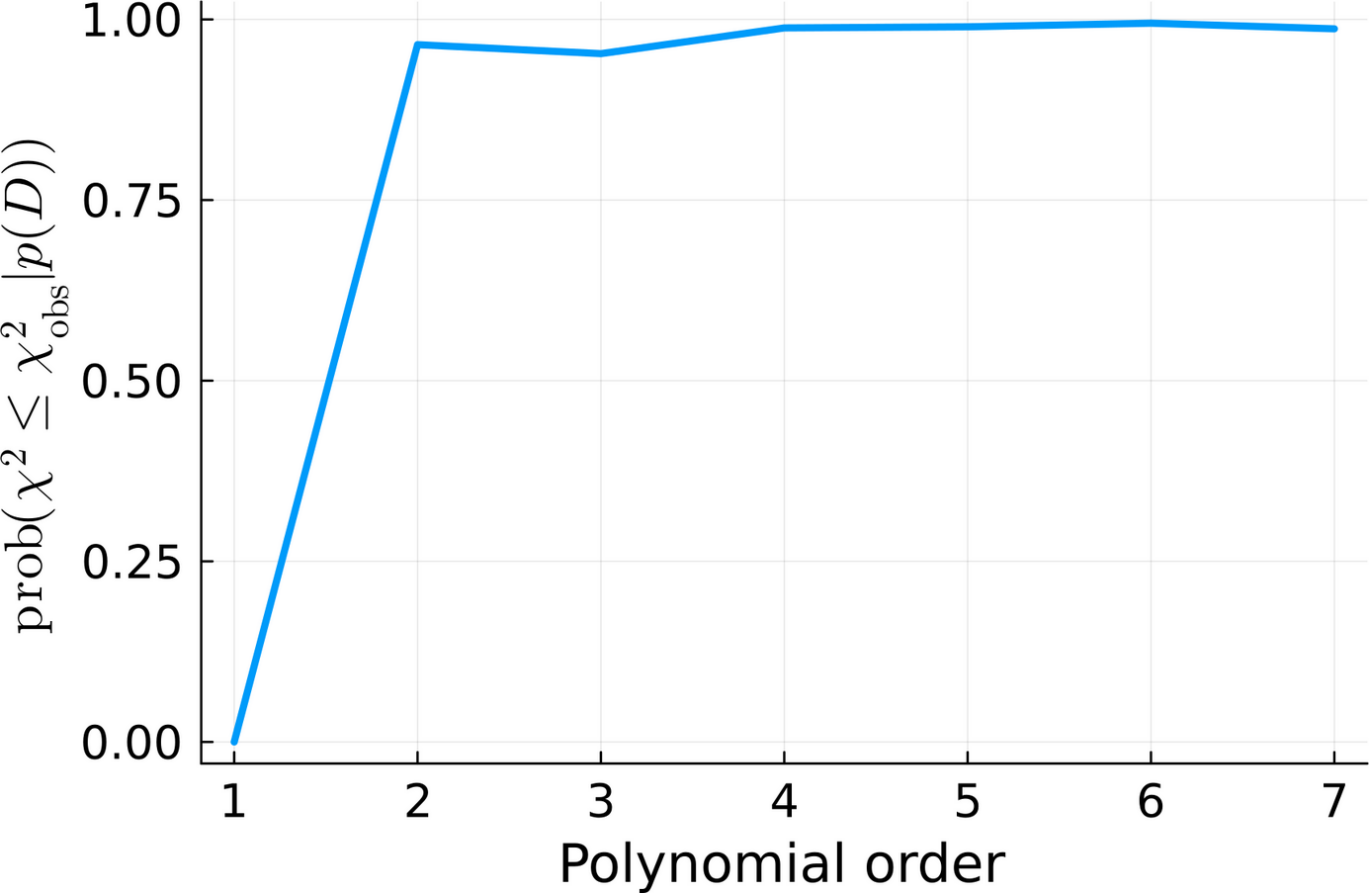
Parameter 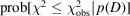 from equation (13) calculated for the χ^2^ = 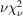 values as in Fig. 16 ▸.

**Table 1:** *D*_eff_ estimated for all the studied observables

Observable	*D*_eff_ (eV atom^−1^)
〈Δ*d*(*t*)〉_vol,real_	4.20
〈*I*_111_(*t*)〉_vol,real_	4.51
〈*I*_311_(*t*)〉_vol,real_	4.28
〈*I*_220_(*t*)〉_vol,real_	4.26
〈*I*_400_(*t*)〉_vol,real_	4.04
〈*I*_331_(*t*)〉_vol,real_	4.02

**Table 2:** Selected data set from experiment (Heimann *et al.*, 2023 ▸)

Case	*E*_0_ (µJ)	*w*_*x*_ × *w*_*y*_ (µm × µm)	λ_e_ (µm)
Tight focus	15	0.173 × 0.196	0.312
